# Tailoring treatment for MALT lymphoma patients: where do we stand now?

**DOI:** 10.18632/oncotarget.23001

**Published:** 2017-12-06

**Authors:** Peter W. Johnson, Catherine Thieblemont, Emanuele Zucca

**Affiliations:** International Extranodal Lymphoma Study Group (IELSG), Foundation for the Research and Cure of Lymphoma in Ticino, Oncology Institute of Southern Switzerland, Bellinzona, Switzerland

**Keywords:** marginal zone B-cell lymphoma, MALT lymphoma, prognostic index, chlorambucil, rituximab

Whilst the role of involved-field radiotherapy for localized disease is well established in MALT lymphoma, the best approach for the patients requiring systemic treatment remains controversial.

The International Extranodal Lymphoma Study Group 19 (IELSG-19) randomized study is the first, and so far the only, randomized trial of systemic treatment for extranodal marginal zone lymphoma of mucosa-associated lymphoid tissue (MALT lymphoma). The original study design compared chlorambucil alone or in combination with rituximab. The protocol was later amended to add a third treatment arm of rituximab alone. Preliminary results of the 2-arm portion of the study were reported in 2013 [1] and recently the whole study results have been reported in two papers [2, 3].

This study demonstrated that rituximab in combination with chlorambucil has superior efficacy over either chlorambucil or rituximab monotherapy in MALT lymphoma [2]. However, improvements observed in complete remission (CR) rate, event-free survival (EFS, the main study endpoint), and progression-free survival (PFS) did not translate into longer overall survival (OS). With a median follow-up of 7.5 years, the-5 year EFS was 51% with chlorambucil alone, 50% with rituximab alone, and 68% with the combination (hazard ratio, HR=0.54, P= 0.0009). PFS was also significantly better with the combination (P= 0.0119) while 5-year OS approximated 90% in every arm. All treatments were well tolerated and unexpected toxicities were not recorded.

When the study was designed, chlorambucil alone was the standard of care for MALT lymphoma. Although rituximab was known to have significant activity against MALT lymphoma, combination chemo-immunotherapy had never been formally tested. The study enrolled 401 evaluable patients. The sample size was calculated on a projected 20% improvement in EFS, a sufficiently large effect to justify the additional cost of rituximab [1].

It is worth noting that this is the only controlled clinical trial to compare chemotherapy or rituximab alone to the combination. The large improvement in EFS (46% reduction in HR; 95%CI 23- 62%) suggests possible synergy for the combination. As the only randomized study specifically addressing MALT lymphoma, these results can be considered a benchmark for future trials. The similar OS between the arms also provides justification for the use of rituximab alone as initial therapy, to delay or avoid the long term risks of chemotherapy. Conversely, this also supports the use of chlorambucil alone when treatment cost is a key consideration.

In this study, patients with primary gastric localization (a stratification criterion) apparently had better CR rate and EFS than those with MALT lymphoma at other sites, although this unplanned analysis must be interpreted with caution, especially since gastric lymphoma patients in the study more often had stage I disease. The study was underpowered to address the clinical relevance of different anatomic localizations.

In a single-arm phase II study of 57 patients [4, 5], the Spanish GELTAMO group evaluated the combination of bendamustine and rituximab as first line systemic treatment of MALT lymphoma, showing an EFS at 7 years of 88%. Whilst these results appear better than those with chlorambucil-rituximab in IELSG19, several unfavourable clinical features were less common in the GELTAMO study, including B symptoms, involvement of multiple extranodal sites, lymph nodes and bone marrow. This makes it hard to directly compare the results of the two studies.

There is no widely accepted prognostic index for MALT lymphoma. Using stepwise Cox regression, the IELSG19 study database allowed the development of a specific prognostic tool for patients with MALT lymphoma. This demonstrated three features of greatest prognostic significance for EFS, namely age ≥70 years (HR 1.72), Ann Arbor stage III or IV (HR 1.79), and elevated LDH (HR 1.87). The prognostic index (named MALT-IPI) using these three parameters identified groups of low, intermediate and high risk (corresponding to the presence of 0, 1 or ≥2 of these factors, respectively). The 5-year EFS rates in these groups were 70%, 56% and 29%, respectively. The MALT-IPI also discriminated between patients of differing PFS, OS and cause-specific survival, was maintained across the 3 treatment arms and in both gastric and non-gastric patients. Histologic transformation during the IELSG19 trial was reported in 10 patients. Notably, 7 of 10 were in the high risk group, and the other 3 in the intermediate risk group (P=<0.001).

An external validation set of 633 patients was studied by merging three independent cohorts of MALT lymphoma patients. This confirmed the MALT-IPI, confirming its discriminating capacity in a large heterogeneous population of patients, irrespective of baseline features and treatment types, although overall outcomes in the validation cohort were worse than in IELSG-19. Cox regression controlling for the cohort effect further confirmed the validity of the prognostic model. Interestingly, the prognostic validity of the MALT-IPI has subsequently been further confirmed in the GELTAMO study cohort treated with R-bendamustine [5]. The new, MALT-IPI, is a simple, accessible and effective tool to identify patients at risk of poor outcomes. It will help to define individual patient treatment approaches and trial designs for the future.
